# *cis*-Bis(2-sulfidopyridine *N*-oxide)platinum(II)

**DOI:** 10.1107/S1600536808002006

**Published:** 2008-01-25

**Authors:** B. Ravindran Durai Nayagam, Samuel Robinson Jebas, Dieter Schollmeyer

**Affiliations:** a Department of Chemistry, Popes College, Sawyerpuram, Tamilnadu, India; b Department of Physics, Karunya University, Coimbatore 641 114, India; c Institut für Organische Chemie, Universität Mainz, Duesbergweg 10-14, 55099 Mainz, Germany

## Abstract

In the crystal structure of the title complex, [Pt(C_5_H_4_NOS)_2_], the Pt atom is coordinated by two O atoms and two S atoms in a *cis* configuration, forming a distorted square-planar coordination geometry. The mol­ecule exhibits pseudo-*C*_2*v*_ symmetry and is essentially planar, with a maximum deviation from planarity of 0.0124 (2) Å. The dihedral angle between the two pyridine rings is 5.85 (2)°.

## Related literature

For related literature, see: Bovin *et al.* (1992 ▶); Chen *et al.* (1991 ▶); Dyksterhouse *et al.* (2000 ▶); Katsuyuki *et al.* (1991 ▶); Leonard *et al.* (1955 ▶); Lobana & Bhatia (1989 ▶); Lydon *et al.* (1982 ▶); Ohms *et al.* (1982 ▶); Symons & West (1985 ▶); Zhou *et al.* (2005 ▶); Shi *et al.* (1997 ▶).
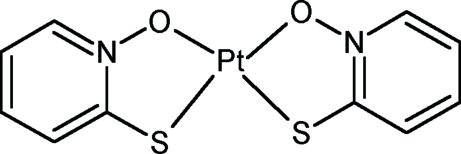


## Experimental

### 

#### Crystal data

[Pt(C_5_H_4_NOS)_2_]
*M* *_r_* = 447.39
Monoclinic, 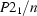

*a* = 6.9832 (3) Å
*b* = 22.3897 (11) Å
*c* = 8.1495 (4) Å
β = 113.572 (3)°
*V* = 1167.87 (10) Å^3^
*Z* = 4
Mo *K*α radiation
μ = 12.36 mm^−1^
*T* = 193 (2) K
0.27 × 0.22 × 0.08 mm

#### Data collection

Bruker Kappa APEXII diffractometer
Absorption correction: multi-scan (*SADABS*; Blessing, 1995 ▶, 1997 ▶) *T*_min_ = 0.051, *T*_max_ = 0.372
25638 measured reflections
2778 independent reflections
2610 reflections with *I* > 2σ(*I*)
*R*_int_ = 0.068

#### Refinement

*R*[*F* ^2^ > 2σ(*F* ^2^)] = 0.054
*wR*(*F* ^2^) = 0.114
*S* = 1.32
2778 reflections
154 parameters
H-atom parameters constrained
Δρ_max_ = 1.73 e Å^−3^
Δρ_min_ = −3.87 e Å^−3^

### 

Data collection: *APEX2* (Bruker, 2006 ▶); cell refinement: *APEX2*; data reduction: *APEX2*; program(s) used to solve structure: *SHELXS97* (Sheldrick, 2008 ▶); program(s) used to refine structure: *SHELXL97* (Sheldrick, 2008 ▶); molecular graphics: *ORTEP2* (Farrugia, 1997 ▶); software used to prepare material for publication: *SHELXL97*.

## Supplementary Material

Crystal structure: contains datablocks global, I. DOI: 10.1107/S1600536808002006/wn2232sup1.cif

Structure factors: contains datablocks I. DOI: 10.1107/S1600536808002006/wn2232Isup2.hkl

Additional supplementary materials:  crystallographic information; 3D view; checkCIF report

## Figures and Tables

**Table:** 

Pt1—O15	2.045 (7)
Pt1—O7	2.052 (7)
Pt1—S16	2.234 (3)
Pt1—S8	2.239 (3)

**Table:** 

O15—Pt1—O7	90.0 (3)
O15—Pt1—S16	86.4 (2)
O7—Pt1—S16	176.3 (2)
O15—Pt1—S8	176.4 (2)
O7—Pt1—S8	86.4 (2)
S16—Pt1—S8	97.13 (10)

**Fig. 1.**
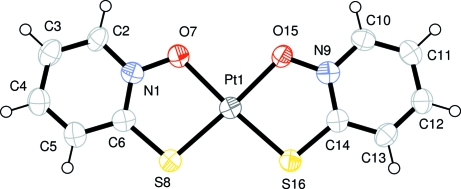
View of the title compound, with displacement ellipsoids drawn at the 50% probability level.

## supplementary crystallographic information

### Comment

N-oxides and their derivatives show a broad spectrum of biological activity, such as antifungal, antibacterial, antimicrobial and antibiotic activities (Lobana & Bhatia, 1989; Symons *et al.*, 1985). These compounds are also found to be involved in DNA strand scission under physiological conditions (Katsuyuki *et al.*, 1991; Bovin *et al.*; 1992). Pyridine N-oxides bearing a sulfur group at the 2-position display significant antimicrobial activity (Leonard *et al.*, 1955). The crystal structure of the title compound in the *trans* configuration has already been reported (Zhou *et al.*, 2005). The crystal structures of *cis*-bis(2-mercaptopyridine N-oxide)palladium and *cis*-bis(2-mercaptopyridine N-oxide)nickel have already been reported (Shi *et al.*, 1997; Chen *et al.*, 1991).

In the title compound, the Pt is coordinated by two O atoms and two S atoms from two mercaptopyridine N-oxide ligands in a *cis* configuration. The coordination geometry is distorted square-planar. The whole molecule exhibits pseudo-C2_v_ symmetry, with a pseudo-C_2_ axis passing through the platinum atom. The average Pt—O and Pt—S distances of 2.048 (7) and 2.236 (3) Å of the title compound are comparable with the values reported in the literature (Dyksterhouse *et al.*, 2000). The mean Pt—S bond length (2.236 (3) Å,) is shorter while the mean Pt—O bond length (2.048 (7) Å) is longer than those reported for the *trans* isomer (Pt—S 2.270 Å; Pt—O 2.007 Å) (Zhou *et al.*, 2005). The C—S bond distances [1.725 (11) and 1.730 (11) Å] are slightly longer than those reported for the uncoordinated thione molecule [1.692 (2)–1.698 (2) Å; Ohms *et al.*, 1982]. The mean C—S bond length (1.727 Å) shows partial double bond character; it is shorter than the normal covalent bond distance of 1.81 (2) Å, for a C—S single bond (Lydon *et al.*, 1982). This further results in the fact that complexes with mercaptopyridine oxide (mpo) ligands have little ability to bridge another metal ion through a sulfur atom to form polynuclear complexes (Shi *et al.*, 1997). The entire molecule is essentially planar, with a maximum deviation from planarity of 0.0124 (2) Å for atom C3. The dihedral angle between the two pyridine rings is 5.85 (2)°.

### Experimental

By heating a mixture of dichlorido(ethane1,2–diamine)platinum(II), Pt(en)Cl_2_ (0.326 g,1 mmol) and 1-hydroxypyridine-2- thione sodium salt (0.298 g, 2 mmol) in 20 ml of water at 338 K with magnetic stirring for 1 h, a red-orange compound was obtained. This was filtered and dried (0.32 g, 80% yield). The compound was dissolved in methanol and allowed to undergo slow evaporation. Fine red crystals were obtained after a week.

### Refinement

H atoms were placed in calculated positions [C—H = 0.95 Å] and refined in the riding model approximation; *U*_iso_(H) = 1.2*U*_eq_ (C). The highest residual electron density peak is 1.724 Å from C13 and the deepest hole lies 1.688 Å from N1.

### Crystal data

**Table:**

[Pt(C_5_H_4_NOS)_2_]	*F*_000_ = 832
*M**_r_* = 447.39	*D*_x_ = 2.545 Mg m^−^^3^
Monoclinic, *P*2_1_/*n*	Mo *K*α radiation λ = 0.71069 Å
Hall symbol: -P 2yn	Cell parameters from 9973 reflections
*a* = 6.9832 (3) Å	θ = 2.8–28º
*b* = 22.3897 (11) Å	µ = 12.36 mm^−^^1^
*c* = 8.1495 (4) Å	*T* = 193 (2) K
β = 113.572 (3)º	Block, orange
*V* = 1167.87 (10) Å^3^	0.27 × 0.22 × 0.08 mm
*Z* = 4	

### Data collection

**Table:**

Bruker APEXII Kappa-CCD diffractometer	*R*_int_ = 0.068
CCD scan	θ_max_ = 27.9º
Absorption correction: multi-scan(SADABS; Blessing, 1995, 1997)	θ_min_ = 1.8º
*T*_min_ = 0.051, *T*_max_ = 0.372	*h* = −9→9
25638 measured reflections	*k* = −29→29
2778 independent reflections	*l* = −10→10
2610 reflections with *I* > 2σ(*I*)	

### Refinement

**Table:**

Refinement on *F*^2^	H-atom parameters constrained
Least-squares matrix: full	*w* = 1/[σ^2^(*F*_o_^2^) + (0.0153*P*)^2^ + 17.8973*P*] where *P* = (*F*_o_^2^ + 2*F*_c_^2^)/3
*R*[*F*^2^ > 2σ(*F*^2^)] = 0.054	(Δ/σ)_max_ = 0.001
*wR*(*F*^2^) = 0.114	Δρ_max_ = 1.73 e Å^−^^3^
*S* = 1.32	Δρ_min_ = −3.86 e Å^−^^3^
2778 reflections	Extinction correction: none
154 parameters	

### Special details

**Table:**

Geometry. All e.s.d.'s (except the e.s.d. in the dihedral angle between two l.s. planes) are estimated using the full covariance matrix. The cell e.s.d.'s are taken into account individually in the estimation of e.s.d.'s in distances, angles and torsion angles; correlations between e.s.d.'s in cell parameters are only used when they are defined by crystal symmetry. An approximate (isotropic) treatment of cell e.s.d.'s is used for estimating e.s.d.'s involving l.s. planes.

### Fractional atomic coordinates and isotropic or equivalent isotropic displacement parameters (Å^2^)

**Table:**

	*x*	*y*	*z*	*U*_iso_*/*U*_eq_	
Pt1	0.75198 (6)	0.117494 (18)	0.60862 (6)	0.03763 (14)	
N1	0.5870 (13)	0.1570 (4)	0.2386 (12)	0.0373 (18)	
C2	0.5399 (16)	0.1976 (5)	0.1045 (15)	0.042 (2)	
H2	0.5774	0.2384	0.1308	0.05*	
C3	0.4376 (18)	0.1791 (6)	−0.0694 (17)	0.050 (3)	
H3	0.4097	0.2066	−0.165	0.059*	
C4	0.3746 (18)	0.1195 (6)	−0.1053 (16)	0.048 (3)	
H4	0.3007	0.1064	−0.2251	0.058*	
C5	0.4207 (16)	0.0803 (5)	0.0340 (15)	0.042 (2)	
H5	0.3772	0.0399	0.0102	0.05*	
C6	0.5318 (17)	0.0990 (5)	0.2124 (15)	0.039 (2)	
O7	0.6952 (11)	0.1788 (3)	0.4073 (10)	0.0380 (15)	
S8	0.5946 (4)	0.05080 (12)	0.3916 (4)	0.0413 (6)	
N9	0.9639 (13)	0.1639 (4)	0.9685 (12)	0.0378 (19)	
C10	1.0646 (17)	0.2052 (5)	1.0962 (16)	0.042 (2)	
H10	1.0885	0.2441	1.0617	0.051*	
C11	1.1311 (17)	0.1915 (5)	1.2724 (16)	0.044 (2)	
H11	1.1986	0.2208	1.3612	0.053*	
C12	1.0994 (17)	0.1342 (5)	1.3215 (15)	0.043 (2)	
H12	1.147	0.1239	1.4445	0.051*	
C13	0.9994 (17)	0.0921 (5)	1.1923 (15)	0.042 (2)	
H13	0.9763	0.053	1.2263	0.051*	
C14	0.9315 (16)	0.1069 (5)	1.0105 (14)	0.038 (2)	
O15	0.8948 (11)	0.1822 (3)	0.7947 (9)	0.0381 (16)	
S16	0.8174 (4)	0.05572 (12)	0.8401 (4)	0.0394 (6)	

### Atomic displacement parameters (Å^2^)

**Table:**

	*U*^11^	*U*^22^	*U*^33^	*U*^12^	*U*^13^	*U*^23^
Pt1	0.0364 (2)	0.0382 (2)	0.0391 (2)	0.00055 (17)	0.01591 (17)	0.00058 (17)
N1	0.032 (4)	0.039 (5)	0.042 (5)	−0.002 (3)	0.017 (4)	−0.001 (4)
C2	0.033 (5)	0.049 (6)	0.039 (6)	0.006 (4)	0.009 (4)	0.011 (5)
C3	0.043 (6)	0.063 (7)	0.046 (7)	0.003 (5)	0.021 (5)	0.013 (6)
C4	0.040 (6)	0.062 (7)	0.041 (6)	0.001 (5)	0.014 (5)	0.000 (5)
C5	0.031 (5)	0.056 (7)	0.041 (6)	−0.003 (5)	0.016 (4)	−0.003 (5)
C6	0.037 (5)	0.041 (5)	0.045 (6)	0.001 (4)	0.023 (5)	0.000 (4)
O7	0.035 (4)	0.043 (4)	0.036 (4)	−0.003 (3)	0.016 (3)	−0.002 (3)
S8	0.0440 (14)	0.0404 (13)	0.0391 (14)	−0.0015 (11)	0.0164 (12)	0.0009 (11)
N9	0.032 (4)	0.041 (5)	0.044 (5)	0.002 (3)	0.020 (4)	0.000 (4)
C10	0.039 (6)	0.039 (5)	0.050 (7)	−0.001 (4)	0.019 (5)	−0.003 (5)
C11	0.037 (6)	0.050 (6)	0.044 (6)	0.000 (5)	0.016 (5)	−0.009 (5)
C12	0.039 (6)	0.052 (6)	0.038 (6)	0.003 (5)	0.016 (5)	−0.001 (5)
C13	0.042 (6)	0.047 (6)	0.043 (6)	0.004 (5)	0.024 (5)	0.007 (5)
C14	0.032 (5)	0.044 (6)	0.040 (6)	0.001 (4)	0.017 (4)	−0.001 (4)
O15	0.038 (4)	0.042 (4)	0.034 (4)	0.000 (3)	0.014 (3)	0.005 (3)
S16	0.0399 (14)	0.0399 (13)	0.0381 (14)	−0.0016 (10)	0.0154 (11)	−0.0001 (10)

### Geometric parameters (Å, °)

**Table:**

Pt1—O15	2.045 (7)	N9—C14	1.364 (13)
Pt1—O7	2.052 (7)	C10—C11	1.356 (16)
Pt1—S16	2.234 (3)	C11—C12	1.389 (16)
Pt1—S8	2.239 (3)	C12—C13	1.376 (16)
N1—C6	1.348 (13)	C13—C14	1.403 (15)
N1—C2	1.357 (13)	C14—S16	1.730 (11)
N1—O7	1.367 (11)	C2—H2	0.9500
C2—C3	1.372 (16)	C3—H3	0.9500
C3—C4	1.399 (17)	C4—H4	0.9500
C4—C5	1.367 (16)	C5—H5	0.9500
C5—C6	1.410 (15)	C10—H10	0.9500
C6—S8	1.725 (11)	C11—H11	0.9500
N9—C10	1.359 (14)	C12—H12	0.9500
N9—O15	1.363 (11)	C13—H13	0.9500
			
O15—Pt1—O7	90.0 (3)	C10—N9—C14	122.1 (10)
O15—Pt1—S16	86.4 (2)	O15—N9—C14	121.0 (9)
O7—Pt1—S16	176.3 (2)	C11—C10—N9	120.6 (10)
O15—Pt1—S8	176.4 (2)	C10—C11—C12	119.2 (11)
O7—Pt1—S8	86.4 (2)	C13—C12—C11	120.2 (11)
S16—Pt1—S8	97.13 (10)	C12—C13—C14	120.0 (10)
C6—N1—C2	123.8 (10)	N9—C14—C13	117.8 (10)
C6—N1—O7	120.7 (9)	N9—C14—S16	119.2 (8)
C2—N1—O7	115.4 (9)	C13—C14—S16	122.9 (9)
N1—C2—C3	119.2 (11)	N9—O15—Pt1	115.1 (6)
C2—C3—C4	119.6 (11)	C14—S16—Pt1	98.1 (4)
C5—C4—C3	119.3 (11)	N1—C2—H2	120.0
C4—C5—C6	121.0 (11)	C3—C2—H2	120.0
N1—C6—C5	117.0 (10)	C2—C3—H3	120.0
N1—C6—S8	120.6 (8)	C4—C3—H3	120.0
C5—C6—S8	122.4 (9)	C3—C4—H4	120.0
N1—O7—Pt1	114.7 (6)	C5—C4—H4	120.0
C6—S8—Pt1	97.5 (4)	C4—C5—H5	119.0
C10—N9—O15	116.8 (9)	C6—C5—H5	120.0
